# Effect of glass shape on the pouring accuracy of liquid volume

**DOI:** 10.1371/journal.pone.0204562

**Published:** 2018-10-23

**Authors:** David M. Troy, Angela S. Attwood, Olivia M. Maynard, Nicholas E. Scott-Samuel, Matthew Hickman, Andy Woods, Marcus R. Munafò

**Affiliations:** 1 Bristol Medical School: Population Health Sciences, University of Bristol, Bristol, United Kingdom; 2 MRC Integrative Epidemiology Unit (IEU) at the University of Bristol, Bristol, United Kingdom; 3 UK Centre for Tobacco and Alcohol Studies, University of Bristol, Bristol, United Kingdom; 4 School of Psychological Science, University of Bristol, Bristol, United Kingdom; 5 Crossmodal Research Laboratory, Department of Experimental Psychology, University of Oxford, Oxford, United Kingdom; University of Portsmouth, UNITED KINGDOM

## Abstract

**Background:**

The shape of glassware may exacerbate or counteract biases in perceived volume, which may lead people to misjudge the pouring of alcoholic drinks. The aim of these studies was to investigate the effect of glass shape on the pouring accuracy of liquid volume.

**Methods:**

In Study 1, using an online computerised task, participants (*n* = 211) were asked to pour liquid in glasses in a within-subjects design with factors of glass shape (straight, curved) and requested percentage fullness (10, 20, 25, 30, 40, 50, 60, 70, 75, 80, 90%). Curve estimations were carried out to determine if errors followed a linear or non-linear relationship. In Study 2, in a real world experimental study, participants (*n* = 96) were asked to pour water to the midpoint of pint glasses in a within-subjects design with one factor of glass shape (straight, curved, tulip, inverted). Differences between poured amounts were analysed using one-way repeated measures ANOVA.

**Results:**

In Study 1, participants under-poured in curved glasses compared to straight glasses at all requested amounts. In Study 2, participants under-poured in curved (*p* < 0.001, *dz* = 1.51) and tulip (*p* < 0.001, *dz* = 0.59) glasses compared to straight glasses. Findings were inconclusive as to whether or not a difference was present between pourings in inverted and straight glasses. Participants displayed a tendency to under-pour in all glasses relative to requested amounts in both studies.

**Conclusions:**

The shape of glassware appears to influence the pouring accuracy of liquid. Pouring in tulip and curved glasses was more inaccurate compared to straight glasses, possibly due to the height of liquid within the glass and volume changing in a non-linear relationship.

## Introduction

Alcohol consumption is a leading cause of ill health and premature death in the world [1]. While effective interventions targeting price [2] and availability [3] exist, alternative approaches that do not directly restrict consumer choice may be more acceptable. One such approach is altering the presentation of objects in settings where alcohol consumption occurs [4]. Changing certain properties of glassware such as the shape [5] and adding volume markings [6] to aid more accurate volume judgements may influence alcohol consumption and therefore could be a target for public health intervention.

Perception of volume appears to be influenced by object shape, with individuals focusing on the most salient dimension as they perceive it (height, length or width depending on the object) when making volume judgements. When individuals use height as the primary dimension to inform their volume estimation, a phenomenon known as the “elongation effect” occurs whereby shorter cylinders are perceived to hold less volume than taller cylinders of the same volume [7–10]. However, this effect reduces as the volume of cylinders increases and the effect is also smaller when cylinders vary in height, width and depth rather than along only one of these dimensions [11]. This effect seems to hold true for some alcoholic products; beer bottles are judged to hold more liquid than shorter beer cans of the same volume [12]. Similarly, with glassware people tend to estimate that tall, slender glasses hold more liquid than wide glasses of the same volume.

Glassware appears to influence how people interact with alcoholic drinks; more alcohol is poured into short, wide glasses compared to tall, slender glasses by both students and bartenders when asked to pour a standard measure [13]. Similarly, people pour more into wider wine glasses than narrower wine glasses [14]. One mechanism which may explain this difference is that individuals tend to focus their pouring attention on the height the liquid reaches and insufficiently compensate for the width of the glass [15]. In work looking at the effect of glass size and shape on wine volume judgements, results were broadly consistent with people using the relative fullness of glasses as the salient dimension to judge volume of wine (i.e., the less full the glass, the less volume was perceived; the more full the glass, the more volume was perceived) [16]. A combination of monitoring the height liquid reaches in glassware and the relative fullness of the glass may be employed when estimating poured amounts.

Glass shape appears to influence how alcoholic beverages are consumed. One study [5] has reported that beer (but not lemonade) is consumed more slowly from a straight glass compared to a curved glass. In addition, weak evidence was found for a positive association between degree of error in estimating the midpoint of a straight and curved glass on a computerised task and total drinking time. This suggests a potential association between glassware that promotes less accurate volume judgements and faster alcohol consumption. A subsequent naturalistic [17] study found monetary takings (i.e. an accurate proxy for alcohol consumption) were reduced when beer and cider were served in straight glasses compared to curved glasses. This suggests that straight glasses as well as reducing the speed of consumption may also reduce the amount of alcohol consumption compared to curved glasses.

Other aspects of glassware seem to alter the consumption of alcoholic beverages. Reducing the size of glassware can lead to reductions in alcohol consumption [18, 19] while applying health warnings [20] and volume markings [6] can slow the consumption of alcoholic beverages. Other environmental factors such as louder music appears to be associated with increased alcohol consumption [21] possibly due to raising levels of arousal and/or altering the taste perception of alcohol to be perceived as more sweet [22] which routinely results in increased alcohol consumption [23].

In summary, perceptual biases involved in volume judgements may affect the pouring and consumption of alcohol. Different shapes of glassware may counteract or exacerbate these biases. We therefore aimed to investigate the effect of glass shape on the pouring of liquid in an online and real world environment (we refer to adjusting volume in the online task as “pouring” for the sake of consistency). Using the computerised task in Study 1, participants were asked to pour liquid to eleven volume percentages in straight and curved glasses. In Study 2, participants were asked to pour water to the midpoint of four pint glasses of different shapes (straight, curved, tulip, inverted). In both studies, we hypothesised that pouring would be more accurate in straight compared to shaped glasses.

## Study 1: Methods

### Design and overview

This was an online study measuring the accuracy of pouring liquid volume, using a within-subjects design with factors of glass shape (straight, curved) and requested percentage fullness (10, 20, 25, 30, 40, 50, 60, 70, 75, 80, 90%). Ethics approval was obtained from the Faculty of Science Research Ethics Committee at the University of Bristol (reference: 310108288). Informed consent was obtained from all participants.

### Participants

Participants were recruited through Amazon’s Mechanical Turk website (https://www.mturk.com). Participants were required to be over 18 and have a verified Mechanical Turk account. We did not bar inclusion based on location, level of expertise in completing online tasks represented by the number of approved Human Intelligence Tasks (i.e. single, self-contained task) or approval rate. Two hundred and ten participants were based in the USA, one was based in the UK.

### Materials

**Fig 1:**
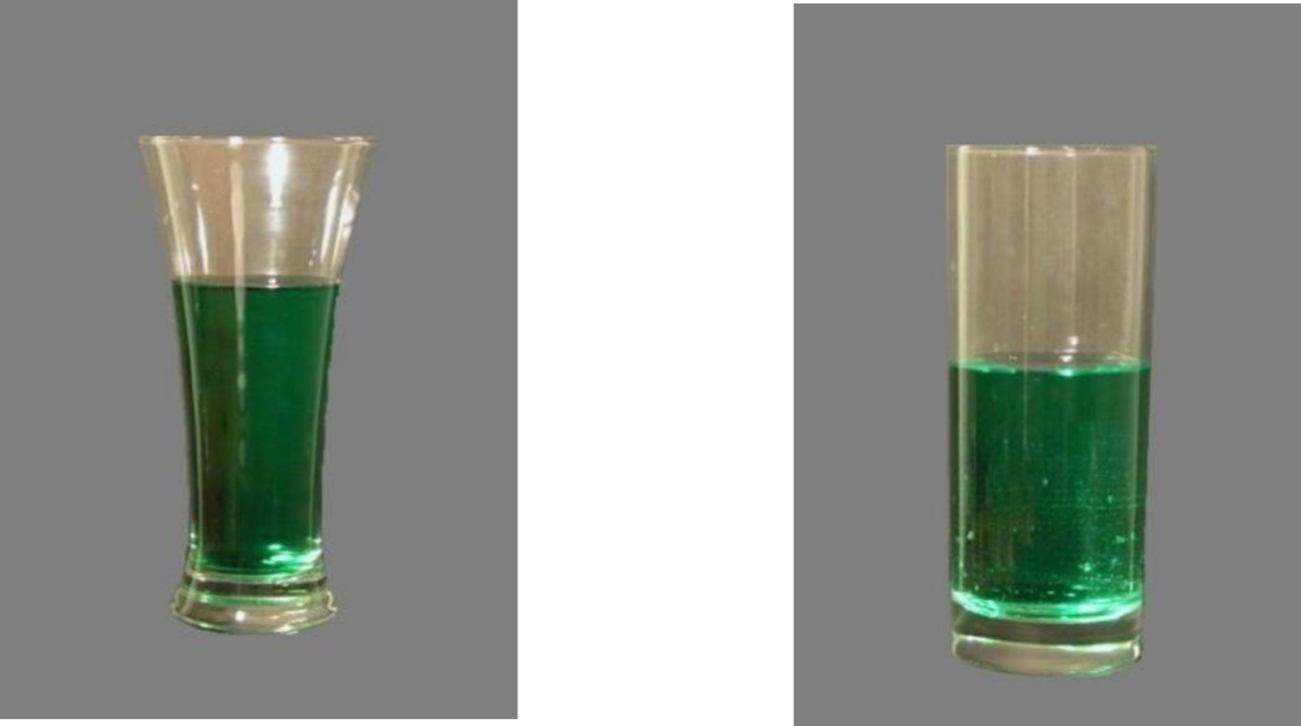
Curved (A) and straight (B) glasses used both poured to 50% capacity.

The curved glass was a pilsner style glass purchased at a local supermarket (Fig 1A). The straight glass was a “highball” style glass designed and supplied by Paşabahçe (Fig 1B). Glass stimuli were generated from sets of photographs taken of these two 12 fluid UK oz (341 ml) glasses using a digital camera (Canon Digital IXUS 70). Each set was a sequence of 60 photographs ranging from an empty (1) to a full (61) glass with liquid added in 60 equal weight increments.

### Procedure

Participants were presented with an information page about the study followed by a page regarding informed consent. After consenting, they were asked to enter demographic characteristics (i.e. age and sex). They were then presented on screen with either a straight or curved glass (Fig 1) and were asked to “pour” designated volume amounts by manipulating the amount of liquid in the glass via their mouse, scroll pad or touch screen. Each participant completed 22 trials (2 glasses × 11 {10, 20, 25, 30, 40, 50, 60, 70, 75, 80, 90%} volume judgements: glass empty at trial start) in random order. On completion of all trials, they were debriefed and given contact details of the experimenter if they wanted to enquire further. The task took approximately seven minutes and participants were reimbursed $1 for their time.

### Statistical analysis

Raw data were converted from scores of 0–60 to millilitres. Data were inspected for outliers via boxplot and were removed if residing three times the interquartile range (IQR) below quartile 1 or above quartile 3. Curve estimations were carried out on the straight and curved glass data to determine if the data followed a linear or non-linear trajectory. Analyses were conducted using IBM SPSS (SPSS Statistics Software Release 23, IBM Corporation). In the absence of a clear basis for estimating a likely effect size, no sample size calculation was carried out prior to data collection. However, our eventual sample size provided 80% power at an alpha level of 5% to detect an effect size of *dz* = 0.19 for the difference in pouring between straight and curved glasses.

The data that form the basis of the results presented here are available from the University of Bristol Research Data Repository (http://data.bris.ac.uk/data/), (to be generated post peer review).

## Results

Participants (*n* = 211; 49% female) were on average 33 years (standard deviation = 12, range = 18 to 65). Data from one participant were excluded for all trials as their responses suggested they did not carry out the experiment as instructed. Otherwise, outlying participant data points were removed at the trial level as the pattern of their data suggested they completed the task as instructed. Outliers removed comprised 0.01% of responses.

**Fig 2:**
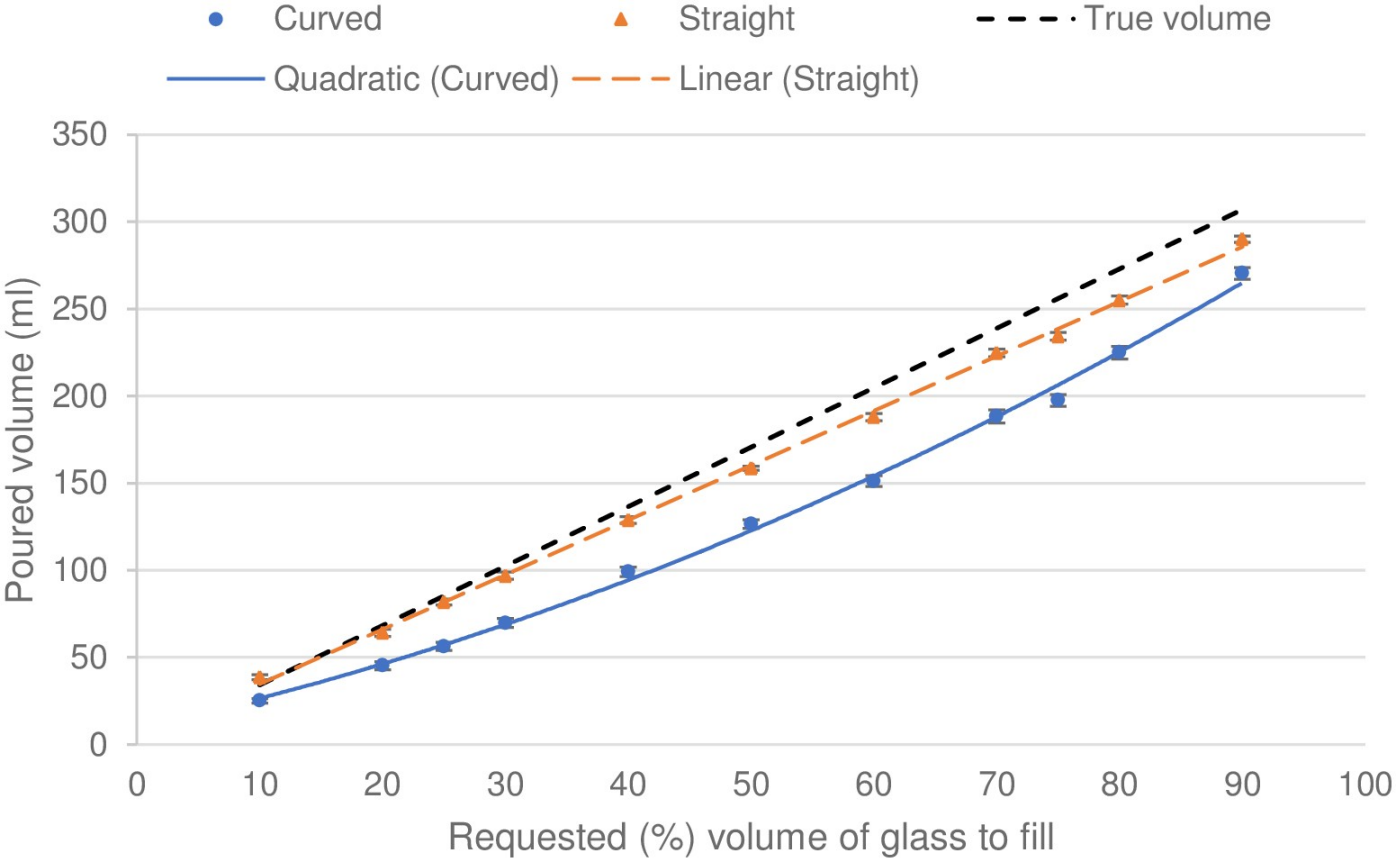
Mean volume poured in millilitres in straight and curved glasses. Error bars represent 95% confidence intervals. Line of best fit has been included for the straight glass data and a curve of best fit has been included for the curved glass data.

Participants under-poured in curved glasses at all requested amounts tested compared to straight glasses (Fig 2). Curve estimations suggested that a linear regression equation best described average data at each requested amount from straight glasses (volume poured in ml = 3.16 + {3.14*requested volume percentage}), while a quadratic regression equation best described average data from curved glasses (volume poured in ml = 9.54 + {1.55*requested volume percentage} + {.01*requested volume percentage^2^}).

## Discussion

Participants under-poured in curved glasses at all points tested compared to straight glasses. This may be explained by individuals using the height of liquid in a glass as a proxy for volume. In straight glasses, the height of the liquid and the volume of the liquid change in a direct, linear relationship resulting in participants pouring more accurately by monitoring the height the liquid reaches in the glass. We next investigated the generalisability of these results in a real world environment using glasses of increased volume capacity of varying shapes.

## Study 2: Methods

### Study design and overview

This study investigated pouring accuracy of liquid volume in different shaped glasses, using a within-subject design with one factor of glass shape (straight, curved, tulip, inverted). Ethics approval was obtained from the Faculty of Science Research Ethics Committee at the University of Bristol (reference: 14061638781). Written informed consent was obtained from all participants. The study protocol was pre-registered prior to data collection on the Open Science Framework (https://osf.io/dbq8q/).

### Participants

Participants aged over 18 were recruited opportunistically from a predominantly staff and student population in the café of the School of Experimental Psychology in the University of Bristol.

### Materials

**Fig 3:**
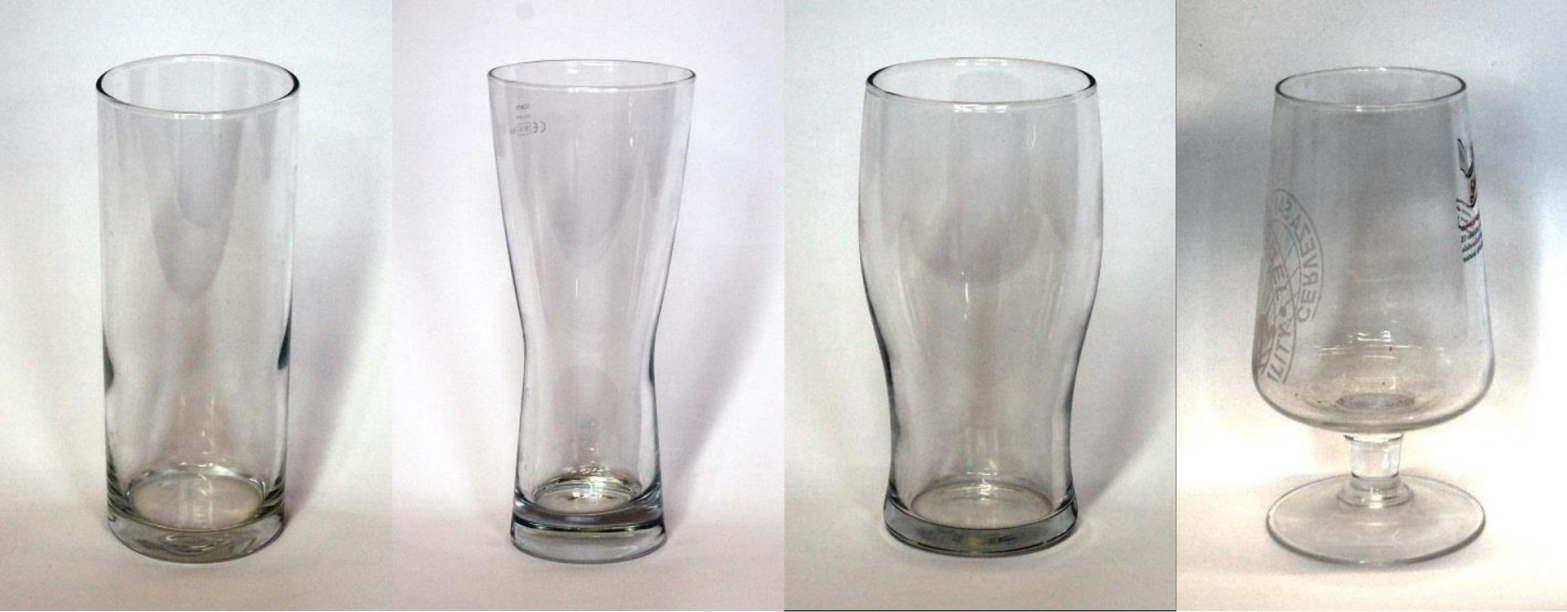
Straight (A), curved (B), tulip (C) and inverted (D) pint glasses.

Four pint glasses (volume: 568 ml) were used (Fig 3). The straight glass was a Geo “highball” style glass supplied and designed by Arcoroc Professional (Fig 3A). The curved glass was a Tokyo style glass supplied and designed by Sahm (Fig 3B). The tulip glass was supplied by Paşabahçe (Fig 3C). The inverted glass was a San Miquel branded stemmed glass supplied by http://www.drinkstuff.com (Fig 3D). A jug filled with water was required for pouring and a 5 ml denominated measuring cylinder was required for measuring. A laptop was used to record volume measurements.

### Procedure

Passers-by and people sitting in the café were asked if they would like to participate in an experiment and were given the opportunity to read a study information sheet and ask questions. Participants were informed prior to pouring that the study was measuring the pouring accuracy of liquid volume. After written informed consent was obtained, participants were asked demographic information (i.e. age and sex), how many units of alcohol they drink a week and if they drink beer. The order in which four glasses were presented included all 24 permutations and an equal number of participants were randomly assigned (using random number assignment software: www.randomizer.org) to each order. Participants were asked to fill glasses presented to them (ordered per randomisation) with water to half the volume the glass could hold. The jug contained enough water (approximately ¾ full) for participants to pour midpoint estimates into the four pint glasses. The jug was not refilled after each pour. After each pouring, the glass was removed from the participant’s sight to avoid comparisons between pourings. When pourings were made in all four glasses, each amount was poured in turn into a measuring cylinder and recorded on an Excel spreadsheet. After testing, participants had the option of entering their email address into a different spreadsheet to enter a draw for a £20 Amazon gift card. Participants were then debriefed as to the purpose of the experiment and final written consent was obtained.

### Statistical analysis

Outliers were inspected via boxplot and were removed if they were three times the IQR below quartile 1 or above quartile 3. Error scores were generated by calculating the differences between the volumes poured into empty glasses and the midpoint volume (284 ml). A one-way repeated measures ANOVA was carried out to determine whether pourings differed in the four glasses. Bonferroni-corrected post-hoc *t* tests were carried out to compare pourings in straight glasses with pourings in other glasses. A between-subjects factor of self-reported units of alcohol consumed in an average week was added to the ANOVA as an exploratory analysis to explore the influence of weekly alcohol consumption on pouring estimations. Analyses were conducted using IBM SPSS (SPSS Statistics Software Release 23, IBM Corporation). We calculated that a sample size of 96 participants would provide 80% power at an alpha level of 5% to detect an effect size of *dz* = 0.29. This effect size was estimated based on exploratory work, which suggested an effect size of *dz* = 1.37 for the difference in average midpoint pouring straight versus curved glasses. We chose a conservative estimate of likely effect size, on the assumption that the effect size observed in our exploratory work was likely to be inflated. Comparisons between tulip versus straight and inverted versus straight glasses were exploratory.

The data that form the basis of the results presented here are available from the University of Bristol Research Data Repository (http://data.bris.ac.uk/data/), (to be generated post peer review).

## Results

Participants (*n* = 96; 65% female) were on average 23 years old (*SD* = 9, range = 18 to 63) and drank an average of 11 units of alcohol a week (*SD* = 13, range = 0 to 80), with 59% reporting that they consumed beer. No outlying data were detected.

**Fig 4:**
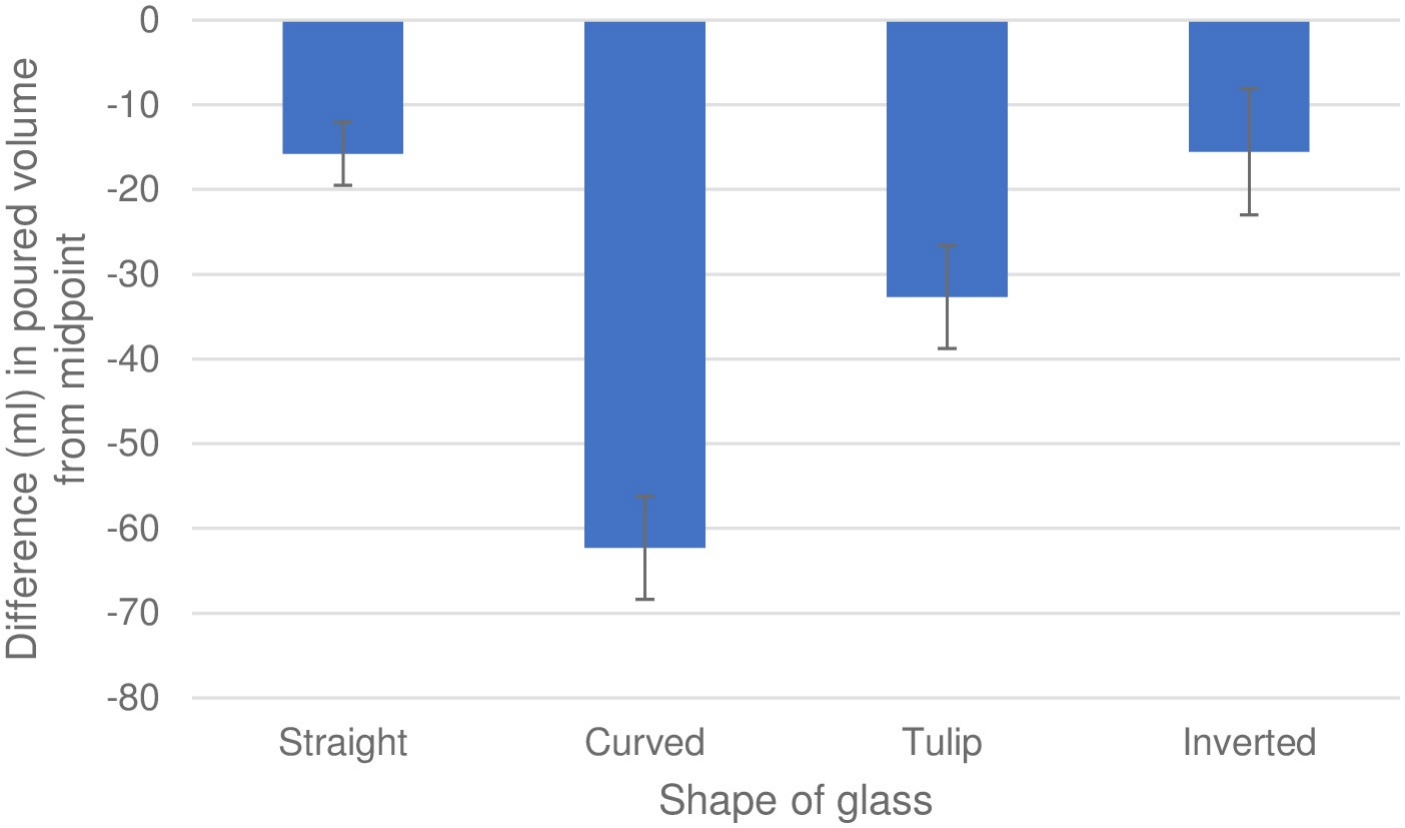
Mean differences in volume poured in millilitres (poured volume in empty glass minus midpoint volume). Error bars represent 95% confidence intervals.

Mauchly’s test indicated that the assumption of sphericity had been violated for the main effect of glass, χ^2^ = 44.99, *p* < 0.001; therefore degrees of freedom were corrected using Greenhouse-Geisser estimates of sphericity (ε = .76). A one-way repeated measures ANOVA suggested strong evidence for a main effect of glass on pourings (*F*_2.29, 217.31_ = 75.51, *p* < 0.001, partial η_p_^2^ = .44). Bonferroni-corrected post-hoc *t* tests revealed strong evidence that pourings differed in curved (*t*_217.31_ = 11.86, *p* < 0.001, *dz* = 1.51) and tulip glasses (*t*_217.31_ = 5.29, *p* < 0.001, *dz* = 0.59) compared to straight glasses (Fig 4). There was no evidence to suggest that pourings in inverted glasses (*t*_217.31_ = .06, *p* = .95, *dz* = 0.01) differed from pourings in straight glasses (Fig 4). There was no evidence for a main effect of number of units consumed in an average week on pourings (*F*_22, 73_ = 0.87, *p* = 0.633, partial η_p_^2^ = .21).

## Discussion

Participants under-poured in tulip and curved glasses compared to straight and inverted glasses. Participants using the height of liquid within glasses as a proxy for volume estimations can broadly explain these findings. Height and volume of liquid changes in a direct, linear relationship in straight glasses while shaped glassware follows a non-linear relationship. Results suggest the more height and volume deviate from a direct, linear relationship in shaped glasses, the more inaccurate pouring becomes. This would explain why pourings in curved glasses were the most inaccurate because the difference in diameter from the narrowest to the widest point of curved glasses was greater than other glasses resulting in an increased overall deviation from a direct, linear relationship. There was no evidence to suggest that weekly alcohol consumption was a good predictor of pouring behaviour. This suggests that regardless of the level of alcohol use, individuals may make the same pouring errors in various glass shapes.

## General discussion

Pourings were closer to the requested amounts (eleven points in Study 1, midpoint in Study 2) in straight glasses compared to curved glasses (Studies 1 and 2) and tulip glasses (Study 2), consistent with our hypothesis. One potential explanation for this is that participants used the height of the liquid as the most salient dimension to estimate volume. Straight glasses may promote more accurate pourings because the height of liquid within the glass and the volume of the liquid changes in a direct, linear relationship.

Interestingly, pourings in the inverted glass were similar in accuracy to straight glasses in Study 2 which was not consistent with our hypothesis. A potential explanation is that the skew of volume distribution within glasses affects pourings. The inverted glass skews the distribution of volume towards the bottom of the glass which results in the true volume midpoint residing below half the height of the glass. Therefore, if participants were aiming to pour liquid to half the height of a glass as a proxy for volume, they would pour more into this glass. Although, it should be noted that participants still under poured compared to true midpoint. The effect of the skew of volume distribution would also explain why curved glasses resulted in the most underestimated pourings as volume distribution is skewed more so than the tulip glass towards the top of the glass. If participants were aiming to pour liquid to half the height of this glass, they would under-pour more so than in the other glasses tested.

Findings from Study 1 suggest an overall trend that the fuller the glass is, the more inaccurate (i.e., underestimated) pourings were in both glasses. This broadly supports other research that suggests that proportions greater than 50% tend to be underestimated [24, 25]. However, accuracy improved in the curved glass as liquid neared 100% (i.e., a full glass). This could be due to a different heuristic being employed by participants when actively pouring to different proportions as opposed to static judgements of proportions. One possible heuristic at work towards the top of glassware could be using the top of the glass as a reference point when making judgements above 75%.

What participants perceived as a full glass may also have influenced performance between 75% and 90% in Study 1. It was not explicitly stated that 100% was deemed as full. If some participants routinely leave some space at the top of a glass when pouring, this mental anchor could have influenced them to pour less near the top of the glass which may partly explain underestimations of volume in this area. Similarly, in Study 2, participants may not have treated a full glass as being 100% full given that the head of a beer would occupy a space at the top of the glass. This mental association may partly explain the underestimation of true midpoint volume in all four glasses. Another factor that may have affected pourings in Study 2 was the amount of water in the jug at the beginning of each trial. The relative fullness of wine glasses has been used to broadly explain volume estimations [16], therefore participants may have used the relative fullness of the jug to inform their pourings. However, the randomisation of the presentation of glasses should have minimised the effect of this extraneous factor.

It is possible the pouring biases seen in these studies may translate to changes in consumption of alcoholic beverages. More accurate pouring in straight and inverted glasses may result in slower consumption in line with other research in laboratory [5] and naturalistic settings [17] which have suggested that glassware that promotes more accurate volume judgements can lead to slower consumption of alcoholic beverages. However, this has to be balanced with more volume being poured into these glasses per pour (assuming the glass is filled to less than full). It may also be the case that shaped glasses that result in the under-pouring of alcoholic beverages may also be an effective health promotion intervention. However, consumption is likely to be more rapid from these glasses. Further studies need to examine the speed of consumption and overall intake of multiple alcoholic beverages from different shaped glasses. Given that the current trend in the UK is towards more people drinking at home [26], it would seem important to determine which glassware on balance would be more effective in reducing harm from excessive alcohol use. Links to the pouring and consumption of alcoholic beverages are tentative given that these studies did not include alcoholic beverages.

Some limitations should be considered when interpreting these results. First, in Study 1, participants used their own computer, tablet or mobile device to complete the study and these varied in terms of display quality and size which may have differentially affected performance on the task. Second, Study 1 was carried out in an online environment on two dimensional stimuli and performance of participants may not generalise to real world environments although, Study 2 would seem to indicate that performance can generalise to offline environments. Third, performance in both studies may not generalise to the pouring of alcoholic beverages given that previous research has found differing effects of volume perception on the consumption of alcoholic and non-alcoholic beverages [5]. Further studies are needed that examine within-subject pouring of alcoholic and non-alcoholic beverages to determine if pouring behaviours are similar for both beverage categories. Fourth, in Study 2, the inverted glass was branded and had a stem (Fig 1D) which was inconsistent with other glasses. Implications drawn from pourings in this glass should be treated with caution. Fifth, no information on habitual alcohol consumption was gathered in Study 1. Therefore, it was not possible to assess the influence of this variable on pouring behaviour. Sixth, the experimental manipulation was not disguised in either study, therefore demand characteristics cannot be ruled out. Differences in pourings may partially be an artefact of these demand characteristics. Finally, the implications of the findings of these studies are limited by the lack of evidence that perceptual biases can influence alcohol consumption. Some preliminary studies [5, 6, 17] suggest it is possible; however, robust replications are needed to strengthen the evidence that correcting volume perceptual biases can result in the reduction of alcohol consumption in real world settings.

## Conclusions

The shape of glassware appears to influence the pouring accuracy of liquid volume. Straight and inverted glassware seem to assist participants in pouring more accurately than curved and tulip glassware in these studies. Further research is needed to disentangle the different perceptual biases present when pouring alcoholic beverages into differently shaped glassware to establish whether results observed here replicate in naturalistic environments, and to determine their effect on the pouring and consumption of multiple alcoholic beverages in a drinking session. These findings could inform viable interventions to reduce population alcohol use and corresponding alcohol-related harms.
